# The Dirty Dozen Scale: Validation of a Polish Version and Extension of the Nomological Net

**DOI:** 10.3389/fpsyg.2016.00445

**Published:** 2016-03-30

**Authors:** Anna Z. Czarna, Peter K. Jonason, Michael Dufner, Małgorzata Kossowska

**Affiliations:** ^1^Faculty of Philosophy, Institute of Psychology, Jagiellonian University in KrakowKrakow, Poland; ^2^School of Social Sciences and Psychology, Western Sydney UniversityBankstown, NSW, Australia; ^3^Institut für Psychologie, Persönlichkeitspsychologie und Psychologische Diagnostik, Universität LeipzigLeipzig, Germany

**Keywords:** Dark Triad, Dirty Dozen, Machiavellianism, psychopathy, narcissism

## Abstract

In five studies (total *N* = 1300) we developed and validated a Polish version of the Dirty Dozen measure (DTDD-P) that measures the three traits of the Dark Triad, Machiavellianism, psychopathy, and narcissism. We detail the presence and stability of a bifactor structure of the 12 items and present evidence for good internal consistency and test–retest reliability. We examine the nomological network surrounding the Dark Triad and show that both the Dark Triad total score and the subscales have acceptable validity. We also present evidence on the Dark Triad and moral behavior. Dark Triad predicts utilitarian moral choice (e.g., approval for sacrificing somebody's life for the sake of saving others) and this link is mediated by low empathic concern. In total, our results suggest that the Polish Dirty Dozen—*Parszywa Dwunastka*—is valid, stable, and useful for the study of lingering puzzles in the literature.

## Introduction

The *Dark Triad* is a term used to describe a set of three personality traits that have attracted much empirical attention in personality, social, and clinical psychology: Machiavellianism, psychopathy, and narcissism. These constructs are conceived of as independent from each other; yet they share an overlapping core that is represented by disagreeableness, callous manipulation, and a short-term, agentic, and exploitative social strategy (Paulhus and Williams, 2002; Jonason et al., 2009; Furnham et al., 2013). Machiavellianism is characterized by strategic, cold, selfish and manipulative behavior in laboratory and real world settings (Christie and Geis, 1970). Psychopathy (i.e., subclinical), is associated with high impulsivity and reckless antisocial behavior, thrill-seeking along with low empathy and anxiety (Hare, 1996). Narcissism is characterized by an unrealistically positive self-view, feelings of entitlement, and a lack of regard for others (Campbell and Miller, 2011). Given substantial interest in the Dark Triad traits, assessment of these traits is of great importance. Each of these traits already has its own extensive literature. As their conceptual resemblance might cause confusion, it is advisable to measure all three traits simultaneously in studies so as to be able to delineate their mutual and their distinct effects (Paulhus and Williams, 2002; Furnham et al., 2013).

However, a major problem is that the assessment of the three Dark Triad traits with established standard questionnaires is highly time consuming. Participants completing the *MACH-IV* (Christie and Geis, 1970), *Self-Report Psychopathy Scale* (SRP-III; Hare, 1985), and the *Narcissistic Personality Inventory* (NPI; Raskin and Hall, 1979) have to respond to more than 90 items. Especially in large-scale studies where multiple personality traits are assessed such a lengthy assessment procedure is not practicable. In such cases, economic assessment is necessary (e.g., Robins et al., 2001; Gosling et al., 2003). Aiming to solve this problem Jonason and Webster (2010) developed a concise questionnaire to assess the Dark Triad traits, the *Dirty Dozen* scale. The scale consists of 12 items, four for each of the three traits comprising the Dark Triad. A large number of studies have affirmed the utility of the scale (e.g., Jonason et al., 2011; Jonason and Webster, 2012; Rauthmann, 2012; Lee et al., 2013; Carter et al., 2014; Aghababaei et al., 2014) and its measurement properties (Jonason and Luévano, 2013; Jonason et al., 2013a; Webster and Jonason, 2013; Küfner et al., 2014; Tamura et al., 2015). Despite some criticism concerning the content of psychopathy and Machiavellanism subscales, and more nuanced facets of psychopathy (Miller et al., 2012; Furnham et al., 2013; Jones and Paulhus, 2014) the instrument has good and stable psychometric properties (Jonason and Webster, 2010; Jonason et al., 2011; Jonason and Luévano, 2013).

In the current research, we sought to develop, and validate, a Polish version of the Dirty Dozen scale, the *Parszywa Dwunastka*. The *Parszywa Dwunastka* is the first measure of the Dark Triad available in Polish language. The development of a Polish version is warranted to spur interest and further research both in Poland—a country of 38 million people—and in international collaborations. We investigated the psychometric quality of the scale and its nomological network in four studies.

To test whether the *Parszywa Dwunastka* has sound psychometric properties, we analyzed its factor structure using principal component and confirmatory factor analyses and computed two forms of reliability (i.e., internal consistency and test-retest reliability). We expected that *Parszywa Dwunastka* would have the same factor structure as the original Dirty Dozen scale. Recent research (Jonason and Luévano, 2013) has shown that a bifactor model accounts for the variability among the 12 items best. This bifactor model views the shared variance among the 12 items as reflective of a single, global factor and its three specific traits or subscales—Machiavellianism, psychopathy, and narcissism. The bifactor model implies that the latent global Dark Triad factor is, in a sense, a measure of the residual Dark Triad after the variance attributable to the three subscale-based latent factors are removed. Likewise, the three subscale-based latent factors reflect their respective measures after removing the variance attributed to the latent global Dark Triad factor. In terms of reliability, we expected modest to moderate internal consistency coefficients (αs) given the small number of items on each scale (Carmines and Zeller, 1979) and, consistent with prior work (e.g., Jonason et al., 2011; Jonason and Webster, 2012), that the psychopathy measure would have the lowest internal consistency whereas narcissism would have the highest. We also expected that *Parszywa Dwunastka* would have good test–retest reliability.

We assess various forms of validity. We expected the subscales of the *Parszywa Dwunastka* to correlate positively with the longer scales of the respective constructs (i.e., the Narcissistic Personality Inventory, (Raskin and Hall, 1979), MACH-IV, (Christie and Geis, 1970); psychopathy subscale of the Polish Diagnostic Inventory of Personality, DKO, (Sanocki, 1980); while the most traditional scale for measurement of psychopathy in English language has 64 items, in Polish forensic practice this shorter, valid MMPI-based scale is used). To further examine the convergent validity of the scales, we tested correlations between the *Parszywa Dwunastka* and the Big Five personality traits, sociosexuality, aggression, and criminality. We expected all three subscales to correlated negatively with agreeableness (e.g., Paulhus and Williams, 2002) and to correlate positively with sociosexuality, aggressiveness, and criminality (Bushman and Baumeister, 1998; Jonason et al., 2009). We also expected men to score higher on all three scales than women (Jonason et al., 2009, 2010b; Jonason and Webster, 2010; Jones and Paulhus, 2014). Finally, we tested the predictive validity of the *Parszywa Dwunastka* by investigating its relationships with a selfish orientation (Jonason et al., 2010c, 2015), indicators of *fast* life strategy (Jonason et al., 2010a), moral choice, and empathic concern (Campbell et al., 2009; Bartels and Pizarro, 2011; Arvan, 2013).

Aside from validating the *Parszywa Dwunastka* these tests of predictive validity would generate new knowledge about the Dark Triad traits. Part of the surge of the interest in the Dark Triad traits over the last 5 years has been the contention that they might be adaptations that align people toward a particular life history strategy (Jonason et al., 2012b). The traits may reflect biases that lead people to adopt short-term, antagonistic solutions to important adaptive and social tasks (Jonason et al., 2009, 2010a). If this was the case, those high in the Dark Triad traits would need a particular psychological make up to enable the successful exploitation of others. A person who cares about others, who is empathetic, and moral, would be impotent to achieve her/his egoistic goals. Hence, in moral dilemmas, the behavior of people high in the Dark Triad traits should be characterized by high utilitarianism and low empathy. We tested these predictions in the current research.

## Study 1

In Study 1, we examined the reliability, factor structure, and nomological network of the *Parszywa Dwunastka.* In terms of reliability, we expected acceptable to good internal consistencies for the narcissism and Machiavellianism scales and moderate internal consistency for the psychopathy scale (Jonason et al., 2011). Concerning the factor structure, we expected that a bifactor model would fit the data well (Jonason and Luévano, 2013). In this model the shared variance among the items reflects a global factor similarly to the general g factor in research on intelligence. The individual items load on two sorts of latent factors: (a) the three latent factors associated with the Dirty Dozen's three subscales (i.e., Machiavellianism, psychopathy, and narcissism) and (b) a latent factor of the global Dark Triad. The latent factors stay uncorrelated. The bifactor model implies that the latent global Dark Triad factor is, in a sense, a measure of the residual Dark Triad after the variance attributable to the three subscale-based latent factors are removed. Likewise, the three subscale-based latent factors reflect their respective measures after removing the variance attributed to the latent global Dark Triad factor. Concerning the nomological network, we expected each of the three Dark Triad traits to correlate positively with established instruments for the assessment of the respective trait. Furthermore, we cast a wide network of construct, convergent, and predictive validity tests. And last, we expected men to score higher on the Dark Triad traits than women (Jonason et al., 2013b; Jones and Paulhus, 2014).

### Methods

#### Participants and procedure

Three hundred four students (193 women; aged 18–54 years; *M* = 22.24, *SD* = 4.69 from Jagiellonian University in Krakow (Poland) received course credit for completing the online surveys described below. Confidentiality of responses was guaranteed. Once they completed the measures, participants were thanked for their participation^1^.

#### Measures

All participants completed the *Parszywa Dwunastka* (Table 1). The original 12-item English-language version was translated forward to Polish by the first author and then back to English independently by a translator with a background in psychology (Church, 2001). Differences between the original English and the back-translation were discussed, and mutual agreements were made as to the most appropriate translation.

**Table 1:** **Principal Components Analysis using an Equamax Rotation of Parszywa Dwunastka items (with English translation) in Study 1 and Item-Level Temporal Reliability in Study 2**.

		**Study 1 (***N*** = 304)**				**Study 2 (*N* = 116)**
**Item**		***P***	***N***	***M***	***r***	**Item-level α across two time points**
		**Narcissism (α = 0.81)**				
4	Dążę do zyskania prestiżu lub pozycji.	−0.23	**0.47**	0.42	*0.44*	0.76
	*I tend to seek prestige or status.*					
5	Zwykle oczekuję szczególnych względów od innych.	0.01	**0.66**	0.20	*0.50*	0.66
	*I tend to expect special favors from others.*					
6	Chcę, żeby ludzie mnie podziwiali.	0.05	**0.91**	−0.01	*0.47*	0.73
	*I tend to want others to admire me.*					
7	Chcę by inni zwracali na mnie uwagę.	0.08	**0.93**	−0.16	*0.41*	0.68
	*I tend to want others to pay attention to me.*					
		**Psychopathy (**α = **0.64)**				
1	Zwykle nie odczuwam wyrzutów sumienia.	**0.79**	0.04	−0.18	*0.23*	0.68
	*I tend to lack remorse.*					
2	Zwykle bywam nieczuły/-a lub niewrażliwy/-a.	**0.55**	−0.23	0.24	*0.30*	0.70
	*I tend to be callous or insensitive.*					
11	Zwykle nie przejmuję się moralnością moich działań.	**0.66**	0.05	0.17	*0.46*	0.54
	*I tend to be unconcerned with the morality of my actions.*					
12	Mam tendencję do cynizmu.	**0.64**	0.15	0.05	*0.40*	0.81
	*I tend to be cynical.*					
		**Machiavellianism (**α = **0.83)**				
3	Zwykle manipuluję innymi by dostać to co chcę.	−0.04	−0.03	**0.86**	*0.63*	0.76
	*I tend to manipulate others to get my way.*					
8	Mam skłonność do wykorzystywania innych do swoich własnych celów.	0.00	0.04	**0.85**	*0.69*	0.78
	*I tend to exploit others toward my own end.*					
9	Stosowałem/-am oszustwa lub kłamstwa by postawić na swoim.	0.08	−0.08	**0.80**	*0.60*	0.75
	*I have used deceit or lied to get my way.*					
10	Używam pochlebstw by dostać to co chcę.	0.13	0.10	**0.60**	*0.56*	0.74
	*I have use flattery to get my way.*					

Factor loadings ≥|0.50| are boldfaced. M, Machiavellianism, P, psychopathy, N, narcissism; r, corrected item–scale correlation (in italics).

To assess the Big Five personality dimensions, we used a Polish-validated 60-item version of the Big Five Inventory (Zawadzki et al., 1998). Participants were asked to respond how much they felt each item described them using a five-point Likert response format (1 = *strongly disagree*; 5 = *strongly agree*). We summed the corresponding items to create scales to create indices for Extraversion (α = 0.81), Neuroticism (α = 0.89), Conscientiousness (α = 0.84), Openness (α = 0.71), and Agreeableness (α = 0.75).

Machiavellianism was measured with a Polish version (Pilch, 2008) of the 20-item Mach IV (Christie and Geis, 1970). Participants were asked to respond how much they agreed or disagreed with statements such as “It is hard to get ahead without cutting corners here and there” (α = 0.78) using seven-point Likert response format (1 = *completely agree*; 7 = *completely disagree*).

To assess subclinical psychopathy, we used a 10-item psychopathy scale from the Polish Diagnostic Inventory of Personality, DKO (Sanocki, 1980), an MMPI-based measure used in official forensic psychological practice in Poland. Participants responded to statements such as “I happen to abuse alcohol” using a dichotomous response format (*Yes*/*No*). We summed “yes” responses to create an index of subclinical psychopathy (α = 0.69).

Narcissism was assessed with the NPI (Raskin and Hall, 1979). The Polish adaptation of the NPI (Bazińska and Drat-Ruszczak, 2000) consists of 34 items and has a five-point Likert response format (1 = *does not apply to me*; 5 = *applies to me*). Items were summed to create an index of narcissism (α = 0.91). This scale had been constructed based on theory and clinical definitions (just as was done in the case of the original NPI), and validated in multiple independent studies (e.g., Golec de Zavala et al., 2009; Dufner et al., 2013; Czarna et al., 2015; Sorokowski et al., 2015).

Sociosexuality was measured with a validated 7-item Polish version (Szymańska, 2006) of the Sociosexual Orientation Inventory (Simpson and Gangestad, 1991). We *z*-scored each item given differing response formats. We aggregated items to produce a total score (α = 0.83) and facets of sociosexuality: Sociosexual Behaviors (α = 0.79), Sociosexual Attitudes (α = 0.85), one item assessing Sociosexual Desires (Webster and Bryan, 2007).

Aggression was measured using a five-point Likert-type response format (1 = *extremely uncharacteristic of me*; 5 = *extremely characteristic of me*) to the items (e.g., “I am an even-tempered person,” reverse-scored) of the Polish version (Tucholska, 1998) of 29-item Aggression Questionnaire (Buss and Perry, 1992). Items were summed to create a composite aggression measure (α = 0.89) as well as four subscales: Hostility (α = 0.80), Anger (α = 0.81), Physical Aggression (α = 0.82), and Verbal Aggression (α = 0.65).

Criminality was assessed with 16 questions with a dichotomous response format (*Yes/No*). Instructions emphasized confidentiality of participants' responses. Questions concerned minor offenses committed by participants (“Have you ever exceeded the speed limit?”, α = 0.70), fraud and theft (“Have you ever concealed your income from tax office?”, α = 0.75), and violent crimes (“Have you ever assaulted somebody?”, α = 0.65). Affirmative responses were summed for each type of criminality. We also calculated total score (α = 0.90) by summing all affirmative responses.

### Results and discussion

#### Reliabilities and correlations between subscales

Internal consistencies were α = 0.83 for Machiavellianism, α = 0.64 for psychopathy, α = 0.81 for narcissism. Consistent with prior research, the three Dark Triad traits were positively correlated (Table 2).

**Table 2:** **Principal Component Correlation Matrix for Study 1**.

	**Narcissism**	**Machiavellianism**	**Psychopathy**
Narcissism	−	0.39^**^ (0.45^**^)	0.02 (0.11)
Machiavellianism		−	0.42^**^ (0.49^**^)
Psychopathy			–

N = 304. Equamax rotation was used. The zero-order correlations among the unweighted composite measures are shown in parentheses for comparison.

** p < 0.01.

#### Factor structure

**Figure 1:**
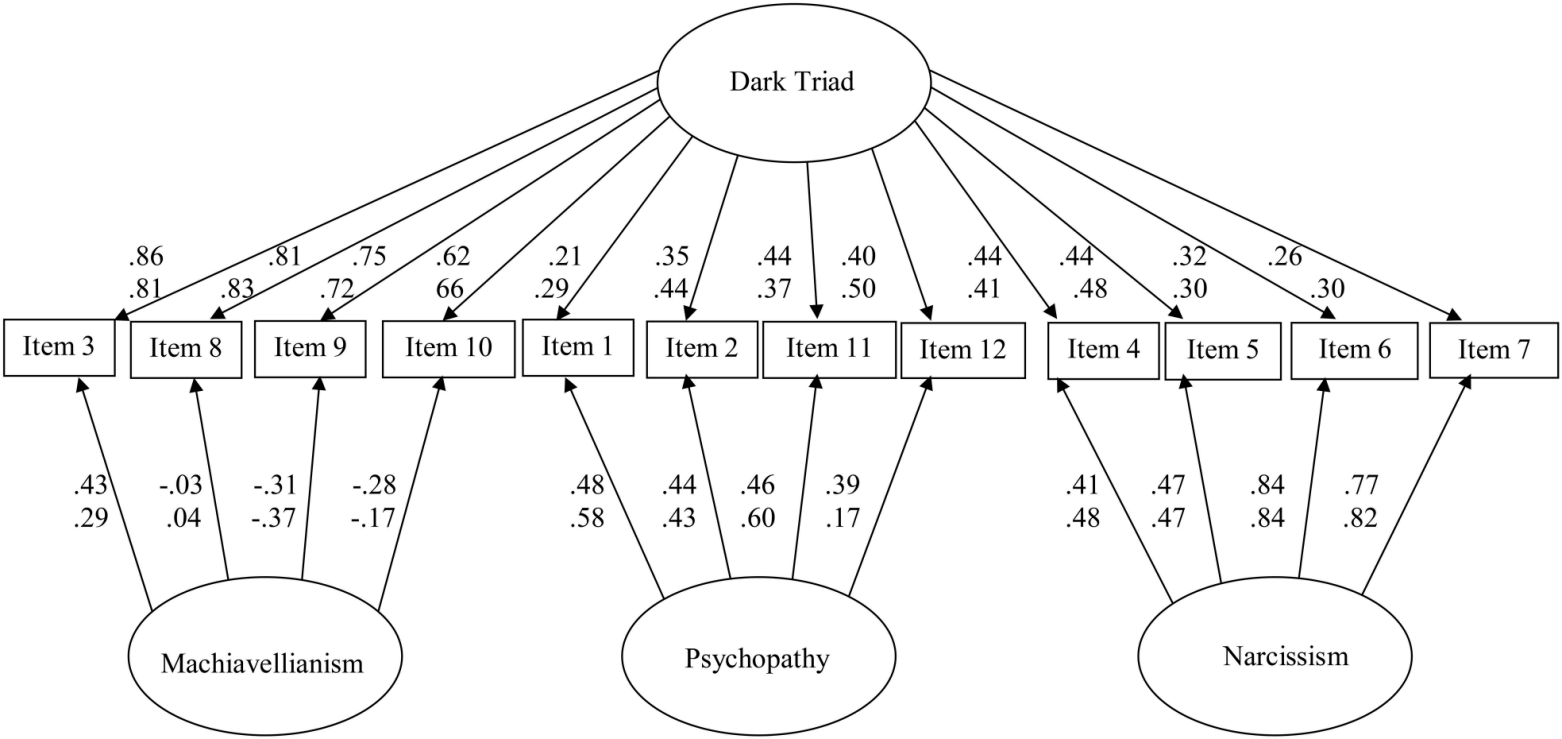
**Bifactor model for the Polish translation of the Dirty Dozen from Study 1 (upper row of numbers) and Study 4 (lower row of numbers)**. Study 1: *N* = 304; $\chi_{\left(42\right)}^{2}$ = 117.65, *p* < 0.01, χ^2^/*df* = 2.80, CFI = 0.94, NFI = 0.91, RMSEA = 0.08, 90% CI [0.06,0.09]). Study 4: *N* = 634; $\chi_{\left(42\right)}^{2}$ = 84.14, *p* < 0.01, χ^2^/*df* = 2.00, CFI = 0.99, NFI = 0.97, RMSEA = 0.04, 90% CI [0.03,0.05]).

We conducted a principal components analysis with an equamax rotation for the *Parszywa Dwunastka*. As predicted, three factors emerged: Machiavellianism, psychopathy, and narcissism (Table 1). They explained 61% of total variance: the three factors accounted for 35, 17, and 8% of the variance, respectively (eigenvalues = 4.2, 2.1, and 1.0). The correlations among these rotated factors are shown in Table 2. When we used confirmatory factor analyses, the bifactor model fit the data well [$\chi_{\left(42\right)}^{2}$ = 117.65, *p* < 0.01, χ^2^/df = 2.80, CFI = 0.94, RMSEA = 0.08; Figure 1, upper numbers].

#### Construct validity

We next tested the convergent and discriminant validity of the *Parszywa Dwunastka* by correlating all three scales with the standard measures of the Dark Triad traits, Big Five traits, sociosexual orientation, aggression and criminality. Despite that the measure of criminality that we used does not exhaust all possible crime types (and further research might elucidate more differences between crime types), the results suggest differential proclivity to different types of crime in dark personalities. Of note, Machiavellianism produced a significantly higher correlation with fraud and theft as compared to the correlation of subclinical psychopathy with fraud and theft (z = 2.16, *p* = 0.016). The two traits did not differ significantly with regard to their associations with the remaining criminal offenses. This result corroborates earlier findings concerning low moral development of Machiavellians and psychopaths (Campbell et al., 2009). Yet, because fraud and theft require more skill and deliberation than impulsive violent crimes, this result also remains in line with conceptualizations of Machiavellianism that underline its strategic deliberate and manipulative orientation and motivation by instrumental or material gain (Jones and Paulhus, 2014). Table 3 (top panel) shows a consistent pattern of convergent and discriminant validity among the *Parszywa Dwunastka* components, with the on-diagonal correlations (validity diagonals) being stronger than the off-diagonal correlations with two exceptions. First, the 20-item measure of Machiavellianism correlated slightly more strongly with the psychopathy scale of the *Parszywa Dwunastka (r* = 0.50) than it did with our concise measure of Machiavellianism (*r* = 0.47). Second, the DKO measure of psychopathy correlated slightly more strongly with the *Parszywa Dwunastka* measure of Machiavellianism (*r* = 0.21) than it did with our concise measure of psychopathy (*r* = 0.20). In neither case, however, was the difference between these dependent correlations statistically significant.

**Table 3:** **Convergent, construct, predictive, and discriminative validity tests for the *Parszywa Dwunastka***.

**Variable**	***Parszywa Dwunastka***			
	**Narcissism**	**Machiavellianism**	**Psychopathy**	**Total**
	**Study 1 (***N*** = 304)**			
Narcissism (34-item NPI)	0.55^**^ (0.64^**^)	0.35^**^ (0.40^**^)	0.14^*^ (0.19^*^)	0.47^**^ (0.54^**^)
Machiavellianism (20-item Mach IV)	0.19^**^ (0.24^**^)	0.47^**^ (0.58^**^)	0.50^**^ (0.71^**^)	0.51^**^ (0.64^**^)
Psychopathy (10-item DKO–subscale)	0.05 (0.07)	0.21^**^ (0.28^**^)	0.20^**^ (0.30^**^)	0.20^**^ (0.27^**^)
**Big Five traits**				
Extraversion	0.20^**^	0.06	−0.16^**^	0.06
Agreeableness	−0.21^**^	−0.48^**^	−0.52^**^	−0.52^**^
Conscientiousness	0.01	−0.11	−0.20^**^	−0.13^*^
Neuroticism	0.12^*^	−0.04	−0.14^*^	−0.03
Openness	0.04	−0.09	0.01	−0.02
**Sociosexual Orientation**	0.15^**^	0.26^**^	0.30^**^	0.31^**^
Sociosexual Attitudes	0.15^*^	0.26^**^	0.41^**^	0.35^**^
Sociosexual Behaviors	0.11	0.20^**^	0.21^**^	0.23^**^
Sociosexual Desires	0.13^*^	0.25^**^	0.21^**^	0.26^**^
**Aggression**				
Physical aggression	0.23^**^	0.47^**^	0.33^**^	0.45^**^
Verbal aggression	0.24^**^	0.36^**^	0.32^**^	0.41^**^
Hostility	0.25^**^	0.26^**^	0.16^**^	0.30^**^
Anger	0.24^**^	0.25^**^	0.09	0.26^**^
Total	0.32^**^	0.43^**^	0.28^**^	0.46^**^
**Criminality**				
Minor offenses	−0.01	0.16^**^	0.15^**^	0.13^*^
Fraud and theft	0.09	0.29^**^	0.17^**^	0.25^**^
Violent crimes	0.14^*^	0.32^**^	0.27^**^	0.32^**^
Crimes – total index	0.11^*^	0.34^**^	0.24^**^	0.31^**^

* p < 0.05;

** p < 0.01.

Construct validity correlations are bolded. Parenthetic correlations are corrected for attenuation from measurement error.

#### Nomological net

The *Parszywa Dwunastka* measures retained a core of disagreeableness (Table 3) with psychopathy and Machiavellanism showing highest associations with it. High psychopathy was linked to low conscientiousness and narcissism to extraversion. All three traits were correlated with all facets of unrestricted sociosexuality, aggression, and proclivity for crime. These results support the validity of the *Parszywa Dwunastka*.

#### Sex differences

Men scored higher than women on the *Parszywa Dwunastka* scales of Machiavellianism, *M*_*f*_ = 2.21, *SD*_*f*_ = 0.85, vs. *M*_*m*_ = 2.61, *SD*_*m*_ = 1.04, *t*_(302)_ = −3.48, *p* < 0.01, *d* = −0.44, psychopathy, *M*_*f*_ = 2.11, *SD*_*f*_ = 0.72, vs. *M*_*m*_ = 2.52, *SD*_*m*_ = 0.87, *t*_(302)_ = −4.50, *p* < 0.01, *d* = −0.54. Men and women were equivalent in narcissism, *M*_*f*_ = 3.19, *SD*_*f*_ = 0.90, vs. *M*_*m*_ = 3.17, *SD*_*m*_ = 0.99, *t*_(302)_ = 0.16, *d* = 0.02.

## Study 2

Thus, far we have shown that the *Parszywa Dwunastka* has good structural properties and is valid. However, the question remains as to whether scale scores are temporally stable. In Study 2, we tested the test–retest reliability of the *Parszywa Dwunastka* and its three component scales. Furthermore, we examined the factor structure and tested measurement invariance across the two time points.

### Method

#### Participants and procedure

One hundred thirty-six part-time students (83 women; aged 18–48 years, *M* = 24.4, *SD* = 6.6) from two universities located in Krakow participated in the study. Each participant was asked to complete the measure twice in class with a 2-week interval between assessments. The items were administered in the one fixed order across the entire study. One hundred sixteen participants (85%) completed both assessments (71 women; mean age = 24.44, *SD* = 6.65). Once they completed the measures, participants were debriefed and thanked for their participation.

#### Measures

We used the *Parszywa Dwunastka* as above. It showed satisfactory internal consistency (i.e., Cronbach's α) at both times (overall α_1_ = 0.82; Machiavellianism α_1_ = 0.77; psychopathy α_1_ = 0.60; narcissism α_1_ = 0.78, and overall α_2_ = 0.87; Machiavellianism α_2_ = 0.84; psychopathy α_2_ = 0.67; narcissism α_2_ = 0.86). The subscales were positively intercorrelated at both times: Machiavellianism correlated with narcissism at the first, *r*_(136)_ = 0.42, *p* < 0.01, and the second measurement, *r*_(116)_ = 0.51, *p* < 0.01, and with psychopathy at the first, *r*_(136)_ = 0.69, *p* < 0.01, and at the second measurement, *r*_(116)_ = 0.79, *p* < 0.01. Narcissism correlated with psychopathy at the first, *r*_(136)_ = 0.38, *p* < 0.01 and at the second measurement, *r*_(116)_ = 0.56, *p* < 0.01).

### Results and discussion

#### Test-retest reliability

We examined the test–retest reliability of the *Parszywa Dwunastka* and its three components by correlating Time 1 with Time 2 scores (Table 4, bolded correlations). The test–retest correlation was .79 for the total score and ranged from 0.67 to 0.75 for its three subscales. These results indicated the *Parszywa Dwunastka* had good test–retest reliability.

**Table 4:** **Temporal Correlation Matrix for the Polish version of Dirty Dozen and Its Three Subscales (Study 2)**.

	**Time 2**		
**Time 1**	**Narcissism (α = 0.86)**	**Machiavellianism (α = 0.84)**	**Psychopathy (α = 0.67)**
Narcissism (α = 0.80)	**0.72**	0.33	0.42
Machiavellianism (α = 0.78)	0.39	**0.75**	0.66
Psychopathy (α = 0.62)	0.31	0.63	**0.67**

N = 116. All rs are significant at p < 0.01. Test–retest reliability correlations are bolded.

#### Factor structure and measurement invariance

We first conducted a confirmatory factor analysis (CFA) to evaluate our *Parszywa Dwunastka* items on the dataset from the first and the second measurement separately. Again, the bifactor model [$\chi_{\left(42\right)}^{2}$ = 88.49, *p* < 0.01, χ^2^/df = 2.11, CFI = 0.94, RMSEA = 0.09] showed good fit in both datasets.

We then investigated temporal stability of the bifactor solution by testing measurement invariance on several levels via multiple-group confirmatory factor analysis (MGCFA). In this strategy, nested models are organized in a hierarchical ordering with decreasing numbers of parameters which entails adding parameter constraints one at a time. These increasingly restrictive models are tested in terms of their fit of the data to the model and compared using Δχ^2^ (Steenkamp and Baumgartner, 1998; Vandenberg and Lance, 2000). The MGFCA following this approach is widely accepted to be the most powerful and versatile approach for testing measurement invariance and is also applicable to longitudinal data (Steenkamp and Baumgartner, 1998; Widaman et al., 2010). Having first established model acceptability at each measurement by itself (see above) we proceeded by testing configural invariance (i.e., same form), followed by invariance of factor loadings, invariance of item-level intercepts and invariance of structural covariances. We did not explore the invariance of item residuals as this is generally considered to be an overly restrictive test with limited applied/practical value (Byrne, 1998).

The bifactor model was first tested for configural invariance and achieved acceptable model fit [Equivalent form model: $\chi_{\left(84\right)}^{2}$ = 147.63, *p* < 0.01, χ^2^/df = 1.76, CFI = 0.94, RMSEA = 0.06], indicating that the *Parszywa Dwunastka* had the same factor structure at both measurements. Next, in a test of metric invariance, all factor loadings were constrained to be equal across groups [Equivalent loadings model: $\chi_{\left(104\right)}^{2}$ = 170.55, *p* < 0.01, χ^2^/df = 1.64, CFI = 0.94, RMSEA = 0.05]. Comparing the unconstrained (i.e., Equivalent form model) and the constrained (i.e., Equivalent loadings model) models yielded an insignificant result [$\Delta \chi_{\left(20\right)}^{2}$ = 22.92, *p* = 0.293, NFI = 0.02, TLI = −0.02], which confirms metric (weak) invariance or, in other words, measurement equivalence in factor loadings. We then extended the constraints to include equivalence in item-level intercepts across measurements [i.e., Equivalent intercepts model: $\chi_{\left(116\right)}^{2}$ = 179.68, *p* < 0.01, χ^2^/df = 1.55, CFI = 0.94, RMSEA = 0.05]. Comparison of this model with Equivalent loadings model yielded no significant change [$\Delta \chi_{\left(12\right)}^{2}$ = 9.13, *p* = 0.692, NFI = 0.01, TLI = −0.01], confirming scalar (strong) invariance. Finally, we constrained structural covariances to be equivalent across measurements [i.e., Equivalent covariances model: $\chi_{\left(120\right)}^{2}$ = 184.49, *p* < 0.01, χ^2^/df = 1.54, CFI = 0.94, RMSEA = 0.05] resulting in a nonsignificant comparison result (i.e., Equivalent covariances against Equivalent intercepts) [$\Delta \chi_{\left(4\right)}^{2}\;=4.81$, *p* = 0.307, NFI < 0.01, TLI < −0.01]. We thus, found evidence of full configural, metric and scalar invariance across measurements. The results showed that the bifactor model held for both measurements across time: the items exhibited the same configuration of salient and nonsalient factor loadings across measurements (i.e., configural invariance) and the slopes (i.e., factor loadings) and intercepts were the same (i.e., invariant) in both measurements (i.e., metric and scalar invariance).

#### Sex differences

Men scored higher than women on the *Parszywa Dwunastka* scales of Machiavellianism on the first measurement, *M*_*f*_ = 1.97, *SD*_*f*_ = 0.70, vs. *M*_*m*_ = 2.29, *SD*_*m*_ = 0.74, *t*_(134)_ = −2.56, *p* = 0.01, *d* = −0.45, and on the second measurement, *M*_*f*_ = 1.84, *SD*_*f*_ = 0.63, vs. *M*_*m*_ = 2.38, *SD*_*m*_ = 0.74, *t*_(114)_ = −4.19, *p* = 0.01, *d* = −0.80. Similarly, men scored slightly higher than women on psychopathy on the first measurement, *M*_*f*_ = 2.14, *SD*_*f*_ = 0.72, vs. *M*_*m*_ = 2.41, *SD*_*m*_ = 0.79, *t*_(134)_ = −2.01 *p* = 0.05, *d* = −0.36, and the second one, *M*_*f*_ = 2.01, *SD*_*f*_ = 0.69, vs. *M*_*m*_ = 2.32, *SD*_*m*_ = 0.80, *t*_(114)_ = −2.24, *p* = 0.03, *d* = −0.41. Men and women did not differ in narcissism at Time 1, *M*_*f*_ = 2.60, *SD*_*f*_ = 0.83, vs. *M*_*m*_ = 2.60, *SD*_*m*_ = 0.84, *t*_(134)_ = −0.07, *p* = 0.99, *d* = −0.01, or Time 2, *M*_*f*_ = 2.40, *SD*_*f*_ = 0.87, vs. *M*_*m*_ = 2.52, *SD*_*m*_ = 0.88, *t*_(114)_ = −0.75, *p* = 0.46, *d* = −0.14.

## Study 3

In Study 3, we used the *Parszywa Dwunastka*'s to examine the correlations between the Dark Triad with life history strategy indicators. Prior research indicates that Dark Triad traits are related to a fast life strategy (Figueredo et al., 2005, 2006; Jonason et al., 2010a, 2012b, 2013b). Life history theory is essentially an evolutionary theory which predicts that personality traits should cluster in a non-random way as adaptations to solve adaptive tasks in response to the stability or harshness of socioecological conditions encountered in young age. A slow life strategy is related to a secure attachment pattern, supportive communication patterns (Olderbak and Figueredo, 2009), a general psychological inclination for long-term strategizing (Gladden et al., 2009), and long-term mating effort and relationships. Because the Dark Triad is associated with a short-term mating strategy along with a strategic approach to friendship-selection, and a protean approach to social interactions, these traits appear to be part of a *fast* life history strategy geared toward maximizing immediate returns over delayed outcomes as predicted by Life History Theory (MacArthur and Wilson, 1967; Wilson, 1975; Rushton, 2004; Buss, 2009).

### Methods

#### Participants and procedure

A convenience sample of one hundred seventy-six students (109 women; aged 18–63 years, *M* = 27.99, *SD* = 8.22) from various faculties of Jagiellonian University in Krakow and their colleagues completed the online surveys described below. Participants completed the *Parszywa Dwunastka*, a measure of life history strategy, and reported their sex and age. Last, participants were thanked and debriefed.

#### Measures

We used a Polish version of the Mini-*K*, a 20-item measure of life history strategy (Figueredo, 2007; Jonason et al., 2013b). Participants completed the questionnaire by indicating how much they agreed (on a five-point Likert response format: 1 = *not at all*, 5 = *very much*) with a series of statements (in Polish) like, “I can often tell how things will turn out” and “I avoid taking risks.” We averaged these items to create an index of life history strategy (α = 0.76), with larger values indicating a slower life history strategy.

Participants then completed the *Parszywa Dwunastka*. Again, the measure showed satisfactory to good internal consistency (Dark Triad α = 0.85, Machiavellianism α = 0.85, psychopathy α = 0.65, narcissism α = 0.88)^2^.

### Results and discussion

Machiavellianism correlated positively with psychopathy, *r*_(176)_ = 0.48, *p* < 0.01, and with narcissism, *r*_(176)_ = 0.53, *p* < 0.01. Narcissism also correlated positively with psychopathy, *r*_(176)_ = 0.18, *p* < 0.05.

#### Life history strategy

As predicted, the Dark Triad traits were negatively correlated with life history strategy, indicating that people higher on Machiavellianism, *r*_(176)_ = −0.17, *p* < 0.05, psychopathy, *r*_(176)_ = −0.25, *p* < 0.01, and narcissism, *r*_(176)_ = −0.16, *p* < 0.05, endorsed a faster life history strategy.

#### Sex differences

Tests for sex differences revealed men scored higher than women on the *Parszywa Dwunastka* scales of Machiavellianism, *M*_*f*_ = 1.76, *SD*_*f*_ = 0.74, vs. *M*_*m*_ = 2.19, *SD*_*m*_ = 1.00, *t*_(174)_ = −3.30, *p* < 0.01, *d* = −0.52, psychopathy, *M*_*f*_ = 1.81, *SD*_*f*_ = 0.62, vs. *M*_*m*_ = 2.34, *SD*_*m*_ = 0.85, *t*_(174)_ = −4.72, *p* < 0.01, *d* = −0.74. The sexes were equivalent in narcissism, *M*_*f*_ = 2.73, *SD*_*f*_ = 1.02, vs. *M*_*m*_ = 2.79, *SD*_*m*_ = 1.11, *t* = −0.33, *d* = −0.05.

## Study 4

We conducted Study 4 to further explore the convergent and discriminant validity of the *Parszywa Dwunastka* by correlating it with measures of social value orientation. Based on existing evidence (Jonason et al., 2010b) those high (vs. low) on the Dark Triad should be more individualistic and competitive. Again we tested for sex differences and examined the factor structure of the *Parszywa Dwunastka*.

### Methods

#### Participants and procedure

Six hundred thirty four students (372 women; aged 19–50 years, *M* = 22.78, *SD* = 3.57) from various faculties of three large Polish technical colleges and universities located in Krakow completed the paper-pencil surveys described below. Once they completed the measures, participants were debriefed and thanked for their participation.

#### Measures

We used the *Parszywa Dwunastka* to assess the Dark Triad. Items were averaged to create indices of Machiavellianism (α = 0.83), psychopathy (α = 0.68), narcissism (α = 0.84).

All participants completed the Triple Dominance Measure (SVO; Van Lange et al., 1997), which is a nine-item measure of social value orientation. In this decomposed game-based measure participants were asked to allocate point amounts to themselves and another person across nine scenarios. Individuals choices reflected not only what they want but what they want a hypothetical other to get, therefore the measure is considered to assess social strategies and is a good indicator of communal orientation. Based on the allocation patterns, three typologies could be identified: prosocial, competitor, and individualist (each participant is identified as prosocial/competitive or individualist based on at least six consistent decisions). Those who failed to make at least six consistent choices and thus did not fit within one of these typologies were labeled as “unclassifiable.” Participants were classified into prosocial (*n* = 282; 44.48%), individualistic (*n* = 200; 31.55%), competitor (*n* = 52; 8.20%), and unclassifiable (*n* = 100; 15.77%) styles.

### Results and discussion

Consistent with prior research, and like in Study 1, the three Dark Triad traits were positively interrelated (Table 2).

#### Factor structure

We used a CFA to test the internal validity of the *Parszywa Dwunastka.* Again the bifactor model [$\chi_{\left(42\right)}^{2}$ = 84.14, *p* < 0.01, χ^2^/df = 2.00, CFI = 0.99, RMSEA = 0.04] fit the data well. Because this sample was our largest across five studies, we present results in Figure 1 (lower row of numbers).

#### Social value orientation

In a single-factor ANOVA we found a significant effect of social value orientation on the Dark Triad composite. Consistent with prior work, Dark Triad scores differed across social value orientation, *F*_(3, 630)_ = 10.81, *p* < 0.01, η^2^ = 0.05. They were significantly lower for those classified as prosocial than those classified as an individualist (*p* < 0.01, η${}_{p}^{2}$ = 0.04) or a competitor (*p* < 0.01, η${}_{p}^{2}$ = 0.03).

#### Sex differences

Men scored higher than women on the *Parszywa Dwunastka* scales of Machiavellianism, *M*_*f*_ = 2.08, *SD*_*f*_ = 0.84, vs. *M*_*m*_ = 2.43, *SD*_*m*_ = 0.93, *t*_(632)_ = −4.92, *p* < 0.01, *d* = −0.40, psychopathy, *M*_*f*_ = 2.00, *SD*_*f*_ = 0.78, vs. *M*_*m*_ = 2.31, *SD*_*m*_ = 0.84, *t*_(632)_ = −4.85, *p* < 0.01, *d* = −0.39, narcissism, *M*_*f*_ = 2.54, *SD*_*f*_ = 0.90, vs. *M*_*m*_ = 2.69, *SD*_*m*_ = 0.89, *t*_(632)_ = −1.96, *p* = 0.05, *d* = −0.16. It might be that for the sex difference in narcissism to be detected, a large sample is required.

## Study 5

Research shows that in sacrificial dilemmas (moral dilemmas where somebody's goods, such as life, may be sacrificed to save other people's goods, e.g., their lives) only 10–36% people select the teleological—utilitarian option (so they approve of sacrificing somebody's life for the sake of saving others; Glenn et al., 2010; Bartels and Pizarro, 2011). In Study 5, we used the *Parszywa Dwunastka* to examine the associations between the Dark Triad and utilitarianism in sacrificial moral dilemmas. Prior research showed that dark personality traits, in particular psychopathy and Machiavellanism were related to low moral development and utilitarian responses to moral dilemmas (Campbell et al., 2009; Bartels and Pizarro, 2011; Koenigs et al., 2012). We expected to replicate this association using *Parszywa Dwunastka*, and we also hypothesized a possible mediation path between the two through low empathic concern. Such a mediation path seemed likely given that previous research findings showed that low empathic concern, characteristic for Dark Triad, predicts utilitarian moral judgment (Gleichgerrcht and Young, 2013). That is, we propose that one potential reason why individuals with high values on the Dark Triad traits make utilitarian moral judgment is that they have low empathic concern for other people.

### Methods

#### Participants and procedure

A convenience sample of **seventy** men (aged 20–33 years, *M* = 23.20, *SD* = 2.24), students and graduates from various universities in Krakow, participated in return for 30 złoty, equivalent of US$ 8 each. They completed an online survey the day before they came to a lab and completed more measures. First, online, participants reported their sex and age and completed the *Parszywa Dwunastka*, and a validated Polish version of Index of Interpersonal Reactivity, plus a number of other dispositional measures which are not relevant for the purpose of current paper^3^. On the next day participants came to lab to complete more measures, including a measure of utilitarianism modeled on Bartels and Pizarro (2011) and Glenn et al. (2010). Last, participants were thanked and debriefed.

#### Measures

Participants completed the *Parszywa Dwunastka*. Again, the measure showed satisfactory to good internal consistencies (Machiavellianism α = 0.83, psychopathy α = 0.74, narcissism α = 0.78).

Emotional and cognitive aspects of empathy were measured with Empathic Sensitivity Questionnaire (ESQ; Kaźmierczak et al., 2007). ESS is a 28-item multidimensional tool based on Davies' theory and modeled on his Index of Interpersonal Reactivity. It uses a 5-point Likert response format. The cognitive aspect of empathy is represented by Perspective Taking subscale (9 items), while its emotional aspect is represented by two subscales: Personal Distress (8 items) and Empathic Concern (11 items). The internal consistencies of the subscales are acceptable (0.80, 0.83, and 0.72, respectively).

The measure of utilitarian and deontological choices in moral dilemmas was modeled on Bartels and Pizarro (2011). We employed three classic stories commonly used in moral cognition research (Baby, Surgery/Transplant, and Spelunkers). After reading the text of a dilemma, each participant was asked to judge whether the protagonist's action was morally permissible; and, participants responded with either a “Yes” or a “No”. Because the action in the scenario always involved hurting someone to produce a greater good (saving many) a Yes response indicated a more utilitarian moral judgment. A second question asked participants to rate their certainty about their answer on a seven-point Likert response scale from “extremely uncertain” to “extremely certain”. For each participant we combined response data and the certainty data into a composite variable by coding “yes” responses as 1 and “no” responses as −1, and multiplying by the certainty ratings (0–7) (cf. Glenn et al., 2010). We calculated the average of these scores across all dilemmas (the overall α = 0.54 for the three-item measure).

### Results and discussion

Machiavellianism correlated positively with psychopathy, *r*_(70)_ = 0.47, *p* < 0.01, and with Narcissism, *r*_(70)_ = 0.43, *p* < 0.01. Narcissism also correlated positively though non-significantly with psychopathy, *r*_(70)_ = 0.17, *p* = 0.16.

#### Utilitarianism

As predicted, the Dark Triad traits associated with utilitarian moral choice. Psychopathy showed stronger relation with utilitarianism, *r*_(70)_ = 0.25, *p* = 0.04, than did narcissism, *r*_(70)_ = 0.20, *p* = 0.09, or Machiavellianism, *r*_(70)_ = 0.16, *p* = 0.18.

We then tested a model of mediation between Dark Triad and utilitarianism via low empathic concern with the PROCESS SPSS macro (Hayes, 2013). Bootstrapping results indicated that the 95% confidence interval of the indirect effect did not contain zero for empathic concern [0.08, 1.0]. Therefore, in line with our hypothesis, empathic concern mediated the effect of Dark Triad and support for sacrificing somebody's life for the sake of saving others.

## General discussion

The current research contributes to the literature by providing the *Parszywa Dwunastka*, a concise measure of the Dark Triad traits in Polish language. In five methodologically distinct studies involving data of more than 1000 participants, we presented evidence for the validity and reliability of the instrument. Despite its brevity, the *Parszywa Dwunastka* had good internal consistency (Carmines and Zeller, 1979) and test–retest reliability. As in the original version of the scale, a bifactor model fit the data well (Jonason and Luévano, 2013). The *Parszywa Dwunastka* had acceptable construct and convergent validity, as indicated by correlations with disagreeableness (Paulhus and Williams, 2002), sociosexuality (Jonason et al., 2009), aggression (Jonason and Webster, 2010), criminality (Hare, 1996), a fast life history strategy (Jonason et al., 2010a), and an individualistic-competitive disposition (Jonason et al., 2010b). Moreover, men consistently scored higher than women on Machiavellianism and psychopathy, while the sex difference in narcissism was not as consistent than for the other two traits. Taken together, these results indicate that the *Parszywa Dwunastka* is an economic, but valid tool for the assessment of the Dark Triad.

Another contribution of the current research is that it sheds more light on the relations between the Dark Triad traits and moral behavior. The findings of Study 4 indicated that individuals who are high in the Dark Triad traits show less prosociality than individuals scoring lower on these traits. In order to minimize impression management we employed hypothetical scenarios instead of measures of self-description. Participants were making decisions about their preferred behavior within a specific situation rather than described themselves. Hence, individuals who are high in the Dark Triad not only describe themselves as immoral, but also tend to declare readiness for immoral decisions when put into a position to do so. Furthermore, the results of Study 5 indicated a positive link between Dark Triad traits and utilitarian decision making and provide a potential explanation for this link. That is, individuals who are high on Dark Triad traits have low empathic concern about other people and this might lead them to take a utilitarian standpoint.

Nevertheless, the Polish Dirty Dozen has a number of limitations. One limitation of the *Parszywa Dwunastka* is that correlations with the original Dark Triad measures were only weak to moderate in size. Similar results were found for the original Dirty Dozen scale (Jonason and Webster, 2010) and for the German translation of the Dirty Dozen scale (Küfner et al., 2014). This finding might be caused by the fact that the original Dark Triad measures assess multiple facets of the respective constructs while the *Parszywa Dwunastka* might only measure the core aspects. However, this possibility requires further research. Moreover, the psychopathy subscale of the *Parszywa Dwunastka* correlated slightly (albeit not significantly) more strongly with long-form Machiavellianism than with long-form psychopathy. This effect was previously shown to be driven by differences in primary and secondary psychopathy (Jonason and Luévano, 2013). Because the Dirty Dozen was designed for brevity, it does not measure such specific facets. Because the measure of psychopathy which we used for the validity tests here was a short questionnaire primarily meant for use in clinical and forensic settings, future validity tests of the psychopathy subscale of Parszywa Dwunastka should use also other instruments, in particular the traditional measure of psychopathy, 64-item SRP-III (Hare, 1985) once it has been translated into Polish language. Also, since our assessment of proclivity for criminal behavior was limited in scope, future studies might use a more exhaustive instrument. Future research would also do well by looking at behavioral outcomes to test predictive validity of the *Parszywa Dwunastka* and also by using indicators of reliability other than Cronbach's alpha, e.g., Greatest Lower Bound or Omega. Furthermore, future research should investigate the association between the Dark Triad traits and utilitarianism in more detail and going beyond low empathic concern in order to elucidate the mechanism underlying this link in its full likely complexity. Nonetheless, for now we suggest that the validity of the Polish Dirty Dozen's psychopathy subscale should be examined by considering a broader pattern of its associations. Consistently with previous research, this subscale showed the strongest associations with low neuroticism, low conscientiousness, and with a fast life strategy (Paulhus and Williams, 2002; Figueredo et al., 2005, 2006; Jonason et al., 2010a, 2012b, 2013b). Altogether, the *Parszywa Dwunastka* provides a satisfactory compromise between precision and efficiency. So far no measure of the Dark Triad has been available to Polish language and this new instrument is needed and has been long awaited. The original measure has been applied in research of a wide variety of topics: from work and counterproductive behaviors through self-presentation, mating strategies, relationship functioning, empathy, pro and antisociality, resource distribution (e.g., Jonason et al., 2012a; Carter et al., 2014; Fox and Rooney, 2015). *Parszywa Dwunastka* is likely to foster further international collaboration on the Dark Triad in a variety of novel research settings.

Brief measures of personality traits have both the costs and benefits. They are costly because some construct breadth and information are sacrificed. They are beneficial because they can eliminate redundant items, save time and effort, and thus reduce participant fatigue and frustration, thus protecting research quality by limiting fatigue-induced errors. Since in large-scale studies, the alternative would often be to drop the construct entirely, the benefits of having a short measure seem self-evident. If brief measures are able to reliability capture the cores aspects of the respective constructs, the benefits outweigh the costs, especially in situations that demand efficiency such as large, and comprehensive personality assessments, experience sampling studies, or longitudinal studies. These measures may push new doors open for research because now the constructs can be assessed in large-scale studies that do not exclusively focus on these traits. Longer more subtle instruments may then be employed to further uncover finer nuances of the detected effects. The results of the present investigation indicate that the Dirty Dozen and its Polish version, the *Parszywa Dwunastka*, represent valuable tools that may be used by researchers to gain a better understanding of the Dark Triad of personality.

## Author contributions

AC and PJ designed studies 1-4, AC designed study 5, AC collected the data. AC, PJ, MD, and MK co-wrote the paper.

## Funding

The present research was supported by a grant awarded to the first author from the National Science Center (DEC-2013/09/D/HS6/02982) in Poland.

### Conflict of interest statement

The authors declare that the research was conducted in the absence of any commercial or financial relationships that could be construed as a potential conflict of interest.

## Footnotes

^1^In all our studies APA ethical standards were adhered to; the studies were carried out in accordance with the research regulations of Institute of Psychology, Jagiellonian University and Bioethical Committee at the Jagiellonian University in Kraków. All subjects gave written informed consent in accordance with the Declaration of Helsinki.

^2^Non-overlapping results from the same study are published in Jonason, Li, and Czarna et al. (2015).

^3^The data come from a yet unpublished psychoendocrinological study using salivatory cortisol and testosterone measurements. The decision to study exclusively men was related to the fact that testosterone is higher in male population. The full list of measures is available on request from the first author.
